# Nonmetastatic cell 1 gene polymorphisms are associated with the clinical progression and prognoses of patients with colorectal cancer

**DOI:** 10.3389/fgene.2025.1485855

**Published:** 2025-05-09

**Authors:** Yabin Liu, Tianliang Bai, Zhenxi Zhang, Yanjun Xu, Dexian Kong

**Affiliations:** ^1^ Department of General Surgery, Fourth Hospital of Hebei Medical University, Shijiazhuang, Hebei, China; ^2^ Department of Gastrointestinal Surgery, Affiliated Hospital of Hebei University, Baoding, Hebei, China; ^3^ Department of General Surgery, Peoples Hospital of Linxi County, Xingtai, Hebei, China; ^4^ Department of General Surgery, Zhangjiakou First Hospital, Zhangjiakou, Hebei, China; ^5^ Department of Endocrinology, Fourth Hospital of Hebei Medical University, Shijiazhuang, Hebei, China

**Keywords:** *NME1*, polymorphism, colorectal cancer, risk, prognosis

## Abstract

**Background:**

Genetic polymorphisms in the nonmetastatic cell 1 gene (*NME1*) are reportedly associated with the risk of various tumors and the prognoses of cancer patients. The aims of this study were to evaluate the contribution of two polymorphisms of *NME1* to the risk of colorectal cancer (CRC) development and the clinical outcomes of CRC patients in a northern Chinese population.

**Methods:**

This study included 453 CRC patients and 464 controls. Genotyping of two polymorphisms (rs2302254 and rs16949649) in the promoter region of *NME1* was performed using the polymerase chain reaction-restriction fragment length polymorphism (PCR-RFLP) method.

**Results:**

The results revealed that the rs2302254 and rs16949649 polymorphisms were not associated with the risk of CRC. However, the rs2302254 TT mutant genotype was associated with an increased risk for advanced clinical stage and lymph node metastasis. Moreover, survival analysis revealed that patients with the homozygous mutant TT genotype of rs2302254 had significantly shorter disease-free survival (DFS) and overall survival (OS) times than patients with the homozygous wild-type CC genotype.

**Conclusion:**

The rs2302254 polymorphism might function as a potential molecular marker for predicting the progression and prognosis of CRC.

## Introduction

Colorectal cancer (CRC) is one of the most common malignant tumors, estimated with 152,810 new cases and 53,010 mortalities occurring in the United States in 2024 (Siegel et al., 2024). Recent years, the incidence rate of CRC has also increased in China (Xu et al., 2023). Despite the high therapeutic success rate for CRC patients with early-stage disease, most patients are diagnosed at clinically intermediate to advanced stages, and the five-year survival rate of CRC patients is only approximately 60% (Cardoso et al., 2022). Metastasis and disease relapse are the major causes of a poor prognosis in CRC patients (Swartjes et al., 2024). Currently, CRC screening methods mainly include colonoscopy and histopathology tests, which exhibit low sensitivity and poor specificity (Onyoh et al., 2019). Therefore, the identification of reliable biomarkers that could help to predict the risk of CRC and monitor the prognosis of CRC patients is essential.

The nonmetastatic cell 1 gene (*NME1*) was first known as a metastasis suppressor gene and was first isolated from murine K-1735 melanoma cell lines (Steeg et al., 1988). This gene product NME1 is the “A” subunit of nucleoside diphosphate (NDP) kinase. Apart from catalyzing the conversion of ADP to ATP in the presence of GTP, NME1 possesses other enzymatic activities, such as histidine protein kinase, serine/threonine-specific protein kinase, and 3′-5′ exonuclease (Roymans et al., 2002; Steeg et al., 2003; Zhang et al., 2011). In addition, NME1 also plays an important role in cell proliferation, differentiation and development, signal transduction, and gene expression (Yu et al., 2021). The reduced expression of NME1 was found to be associated with increased metastatic behavior in various tumors, including breast carcinoma and lung cancer (Zhang et al., 2023; Lu et al., 2024). However, the opposite trend was observed in some other cancers, such as gastric cancer, hepatocellular carcinoma, and neuroblastoma (Xie et al., 2023; Yang et al., 2022; Chicco et al., 2023). Thus, NME1 might play different roles in carcinomas of different origins. In colorectal tumor tissues, the expression of NME1 was shown to be low, and reduced NME1 was associated with higher Dukes stages, poorer differentiation, and positive lymph node metastases in CRC patients (Yang et al., 2016; Lin et al., 2011). Furthermore, CRC patients with NME1-negative tumors had a poorer prognosis than those with NME1-positive tumors (Fu and Chu, 2015). These findings indicate that NME1 might be involved in the formation and progression of CRC.

The human *NME1* lies on chromosome 17q21, and several single nucleotide polymorphisms have been identified within this gene. Among them, two functional polymorphisms, rs2302254 and rs16949649, have been reported to be associated with the risk of developing various tumors and the prognosis of cancer patients. For example, Rajagopal et al. (2020) revealed that the CC genotype frequency of rs16949649 was 13.2% in the breast cancer patients and 8.4% in the control group, and the rs16949649 CC genotype was associated with a significantly increased risk of breast cancer. Li et al. (2012) showed that the rs2302254 TT mutation genotype frequency was 9.3% in the ovarian cancer patients, and the ovarian cancer patients with rs2302254 T/T genotype had a shorter survival time. However, little is known regarding the effects of *NME1* genetic variants on CRC. In this study, we evaluated the contribution of the two polymorphisms of *NME1* to the risk of CRC development and the clinical outcomes of CRC patients in a northern Chinese population.

## Materials and methods

### Study subjects

In this case‒control study, a total of 917 peripheral blood samples from 453 CRC patients and 464 controls were collected consecutively from May 2016 to December 2021 at the Fourth Hospital of Hebei Medical University. All CRC patients were diagnosed via pathology. Patients who had any other previous malignant tumors or who received adjuvant chemotherapy or preoperative radiotherapy were excluded from this study. The controls were recruited from healthy volunteers who participated in general health checks at the Fourth Hospital of Hebei Medical University during the same time-period of case collection. The exclusion criterion for the control subjects was no personal or family history of malignant disease.

After patients underwent surgical treatment for CRC, follow-up was performed from July 2016 to January 2024. The clinical symptoms, serum carcinoembryonic antigen levels, and computed tomography scans were obtained to assess potential relapse of CRC. The survival data of CRC patients were also evaluated. Disease-free survival (DFS) was defined as the time interval between the date of surgery and the date of first disease relapse. Overall survival (OS) was defined as the time interval from the date of study entry to the date of death from any cause.

All study participants were unrelated Han Chinese people who resided in Shijiazhuang and the surrounding areas. Written informed consent was provided by all the recruited subjects according to the Helsinki declaration. This case‒control study was approved by the Ethics Committee of the Fourth Hospital of Hebei Medical University.

### DNA extraction and genotyping

Genomic DNA was extracted from the collected peripheral blood within 1 week using the salting-out method. Genotyping of the two *NME1* polymorphisms was performed with the polymerase chain reaction-restriction fragment length polymorphism (PCR-RFLP) method. The primers for PCR amplification of rs2302254 were 5′-CGC GAA CGA AGG AAG TGA GTC A-3′ (forward) and 5′-GCC GCC AGC ACC CGA AAC-3′ (reverse). The primers for rs16949649 were 5′-CGG CTC CTG ATT CCA TTT TTG TAC-3′ (forward) and 5′-GCT TCT GGG AGG GAT GGG AGT ATA-3′ (reverse). PCR was conducted in a 20 μL volume including 100 ng of DNA template, 200 nM of each primer, 2 μL of 10XPCR buffer, 1 unit of Taq DNA polymerase (TiangeBjotech, Beijing, China), 0.4 μL of 10 mM dNTPs, and nuclease-free water. The cycling conditions for PCR were 94°C for 5 min, followed by 35 cycles of 94°C for 45 s, 63°C for 45 s for rs2302254, 62°C for 45 s for rs16949649, and 72°C for 45 s, with a final elongation at 72°C for 7 min. The amplified PCR products were digested overnight at 37°C with the 5 U restriction enzyme BanII (Sangon Biotechnology Co., Ltd., Shanghai, China) for rs2302254 and HinfI (Sangon Biotechnology Co., Ltd., Shanghai, China) for the rs16949649 polymorphism. The digested products were separated on a 4% agarose gel and visualized under ultraviolet light. In addition, to validate the results of PCR-RFLP, the genotypes in approximately 10% of the samples were detected with direct sequencing method, and the results were 100% concordant.

### Statistical analysis

All the data analyses were performed with SPSS 22.0 (SPSS Inc., Chicago, IL, United States). The fitness of the genotype frequencies to Hardy–Weinberg equilibrium (HWE) was analyzed with a chi-squared test. Comparisons of clinical data between the case and control groups were conducted with Student's *t*-test or a chi-squared test. Differences in genotype distribution between CRC patients and controls were compared with a chi-squared test and Bonferroni’s correction. An unconditional logistic regression model was applied to calculate the odds ratios (ORs) and 95% confidence intervals (CIs). Survival analyses were evaluated with the Kaplan‒Meier (K–M) method, and differences among genotypes were estimated with a log-rank test. Univariate and multivariate analyses were conducted with the Cox proportional hazards regression model. A *P* value less than 0.05 was considered to indicate statistical significance.

## Results

### Characteristics of the study subjects

The mean ages of the CRC patients and controls were 61.53 ± 12.48 years (range 26–86) and 60.91 ± 11.50 years (range 29–81), respectively. There was no significant difference between the two groups with respect to age (*P* = 0.437). In addition, the sex distribution was also similar between the cases and controls (*P* = 0.277). The genotype distributions of rs2302254 and rs16949649 were in line with Hardy-Weinberg equilibrium in the control group (*P* = 0.681 and 0.974, respectively).

### The genotypic frequencies of rs2302254 and rs16949649 polymophisms in patients with CRC and controls

Table 1 summarizes the genotype frequencies of rs2302254 and rs16949649. The frequencies of the rs2302254 CC, CT, and TT genotypes were 54.3%, 36.2%, and 9.5%, respectively, in patients with CRC and 58.0%, 35.3%, and 6.7%, respectively, in controls. No significant difference was detected in the genotype frequencies of rs2302254 between the case and control groups (Table 1). Similarly, the genotype frequencies of the rs16949649 polymorphism did not vary significantly between patients with CRC and controls (Table 1).

**TABLE 1:** The genotypic frequencies of rs2302254 and rs16949649 polymophisms in patients with colorectal cancer and controls.

Group	Controls (%)	Cases (%)	OR (95% CI)	*P*	OR (95% CI)^a^	*P* ^a^
rs2302254 genotype						
CC	269 (58.0)	246 (54.3)	Reference		Reference	
CT	164 (35.3)	164 (36.2)	1.09 (0.83–1.44)	0.527	1.09 (0.82–1.43)	0.557
TT	31 (6.7)	43 (9.5)	1.52 (0.93–2.48)	0.096	1.55 (0.95–2.55)	0.082
rs16949649 genotype						
TT	163 (35.1)	142 (31.3)	Reference		Reference	
TC	222 (47.8)	221 (48.8)	1.14 (0.85–1.53)	0.371	1.14 (0.85–1.53)	0.374
CC	79 (17.0)	90 (19.9)	1.31 (0.90–1.91)	0.162	1.32 (0.90–1.92)	0.153

Abbreviations: OR, odds ratio; CI, confidence interval.

^a^ Adjusted for age and sex.

### Associations of rs2302254 and rs16949649 with the clinicopathological characteristics of CRC patients

Results of the stratified analysis of *NME1* polymorphisms and clinicopathological features of CRC patients are listed in Table 2. The rs2302254 and rs16949649 polymorphisms were not associated with age, sex, or histological grade of CRC patients. However, patients with the rs2302254 TT genotype were at significantly greater risk of having advanced clinical stage and lymph node metastasis than those with the rs2302254 CC genotype (Table 2; *P* = 0.023 and 0.026, respectively).

**TABLE 2:** Association of rs2302254 and rs16949649 with the clinical characteristics of colorectal cancer patients.

Group	rs2302254 C/T			*P*	rs16949649 T/C			*P*
	CC (n, %)	CT (n, %)	TT (n, %)		TT (n, %)	TC (n, %)	CC (n, %)	
Age, years								
<60	93 (50.5)	69 (37.5)	22 (12.0)		64 (34.8)	80 (43.5)	40 (21.7)	
≥60	153 (56.9)	95 (35.3)	21 (7.8)	0.231	78 (29.0)	141 (52.4)	50 (18.6)	0.174
Gender								
Male	146 (54.7)	98 (36.7)	23 (8.6)		83 (31.1)	130 (48.7)	54 (20.2)	
Female	100 (53.8)	66 (35.5)	20 (10.8)	0.744	59 (31.7)	91 (48.9)	36 (19.4)	0.972
Differentiation								
Level 1–2	203 (56.2)	126 (34.9)	32 (8.9)		111 (30.7)	182 (50.4)	68 (18.8)	
Level 3	43 (46.7)	38 (41.3)	11 (12.0)	0.248	31 (33.7)	39 (42.4)	22 (23.9)	0.346
Clinical stage								
I - II	160 (58.6)	94 (34.4)	19 (7.0)		86 (31.5)	137 (50.2)	50 (18.3)	
III - IV	86 (47.8)	70 (38.9)	24 (13.3)	**0.023**	56 (31.1)	84 (46.7)	40 (22.2)	0.573
Lymphatic metastasis								
No	170 (58.0)	102 (34.8)	21 (7.2)		96 (32.8)	144 (49.1)	53 (18.1)	
Yes	76 (47.5)	62 (38.8)	22 (13.8)	**0.026**	46 (28.8)	77 (48.1)	37 (23.1)	0.392
Tumor location								
Left-sided colon	168 (53.2)	118 (37.3)	30 (9.5)		100 (31.6)	149 (47.2)	67 (21.2)	
Right-sided colon	78 (56.9)	46 (33.6)	13 (9.5)	0.732	42 (30.7)	72 (52.6)	23 (16.8)	0.463

*P* values in bold were statistically significant.

### Associations of rs2302254 and rs16949649 with the prognosis of CRC patients

**FIGURE 1:**
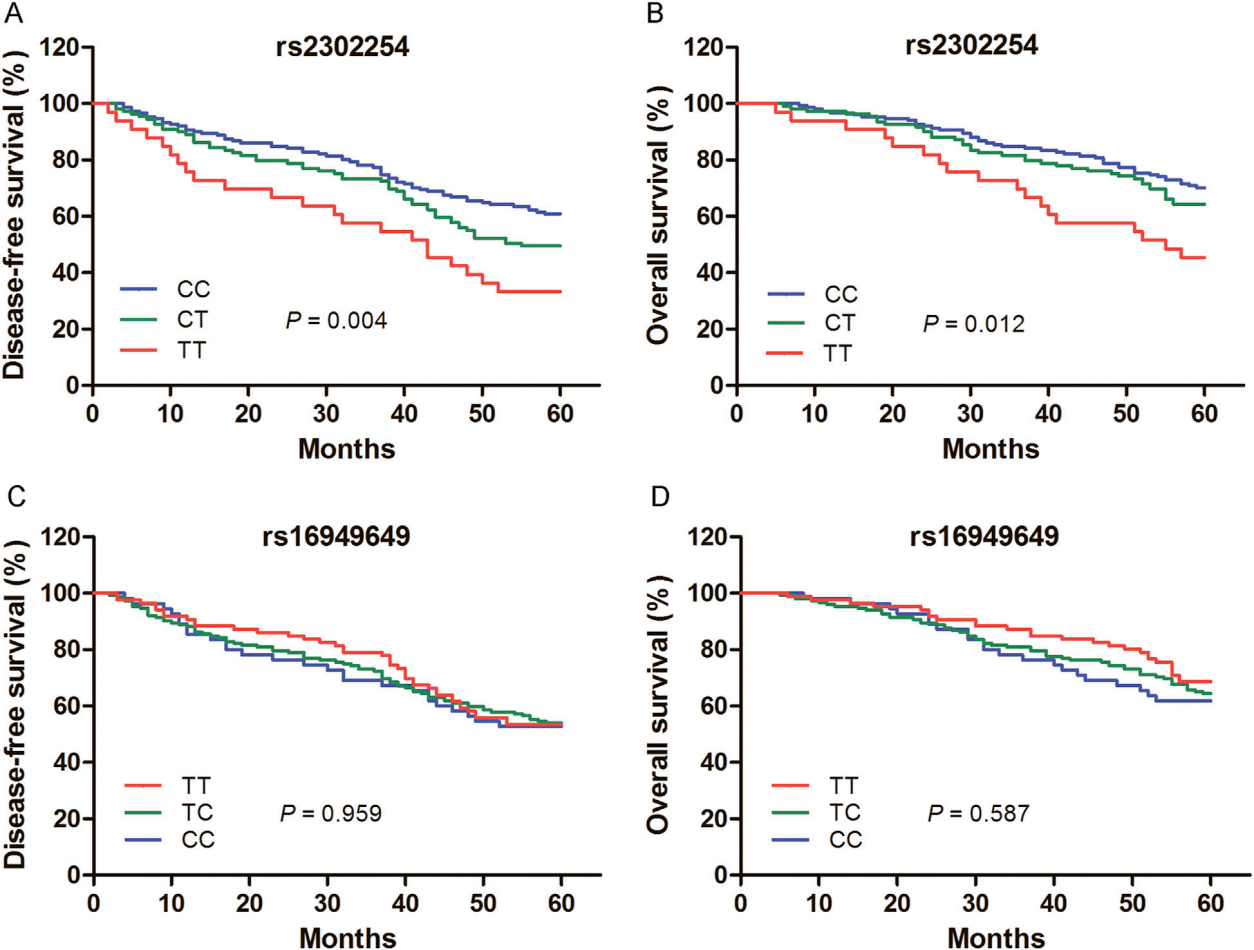
Kaplan–Meier evaluates for the prognosis of colorectal cancer (CRC) patients according to the genotypes of rs2302254 and rs16949649. **(A)** Mean disease-free survival times of CRC patients carrying the rs2302254 CC, CT, and TT genotypes were 48.0, 44.5 and 37.0 months, respectively (*P* = 0.004). **(B)** Mean overall survival times of CRC patients carrying the rs2302254 CC, CT, and TT genotypes were 52.8, 51.1 and 44.9 months, respectively (*P* = 0.012). **(C)** Mean disease-free survival times of CRC patients carrying the rs16949649 TT, TC, and CC genotypes were 46.7, 45.2 and 44.2 months, respectively (*P* = 0.959). **(D)** Mean overall survival times of CRC patients carrying the rs16949649 TT, TC, and CC genotypes were 53.2, 50.8 and 49.7 months, respectively (*P* = 0.587).

Among the 453 CRC patients, 293 were followed for more than 5 years. Kaplan–Meier plots revealed that the rs2302254 polymorphism was significantly associated with the clinical outcomes of CRC patients (Figures 1A,B). Patients with the homozygous mutant TT genotype of rs2302254 had significantly shorter DFS and OS times than patients with the homozygous wild-type CC genotype according to univariate analysis (Tables 3, 4; HR = 2.22, 95% CI = 1.36–3.63, *P* = 0.001; HR = 2.25, 95% CI = 1.30–3.69, *P* = 0.004). The significant association between rs2302254 and CRC patient prognosis was maintained according to a multivariate analysis (Tables 3, 4; HR = 2.09, 95% CI = 1.27–3.46, *P* = 0.004; HR = 2.02, 95% CI = 1.16–3.54, *P* = 0.014). Considering the rs16949649 polymorphism, survival analysis suggested that this genetic variant was not related to the DFS or OS of CRC patients (Figures 1C,D; Tables 3, 4).

**TABLE 3:** Association between *NME1* polymorphisms and the 5-year disease-free survival of colorectal cancer patients.

Genotype	n = 293	No. of recurrence (%)	Univariate model		Multivariate model	
			HR (95% CI)	*P*	HR (95% CI)^a^	*P* ^a^
rs2302254						
CC	151	59 (39.1)	Reference		Reference	
CT	109	55 (50.5)	1.39 (0.96–2.01)	0.078	1.32 (0.91–1.92)	0.146
TT	33	22 (66.7)	2.22 (1.36–3.63)	**0.001**	2.09 (1.27–3.46)	**0.004**
rs16949649						
TT	86	40 (46.5)	Reference		Reference	
TC	152	70 (46.1)	1.03 (0.70–1.52)	0.893	0.97 (0.65–1.45)	0.886
CC	55	26 (57.3)	1.06 (0.66–1.76)	0.775	0.91 (0.54–1.51)	0.709

*P* values in bold were statistically significant.

Abbreviations: HR: hazard ratio; CI: confidence interval.

^a^ Adjusted for age, sex, differentiation, clinical stage, lymphatic metastasis and tumor location.

**TABLE 4:** Association between *NME1* polymorphisms and the 5-year overall survival of colorectal cancer patients.

Group	n = 293	No. of death (%)	Univariate model		Multivariate model	
			HR (95% CI)	*P*	HR (95% CI)^a^	*P* ^a^
rs2302254						
CC	151	45 (29.8)	reference		reference	
CT	109	39 (35.8)	1.25 (0.82–1.92)	0.306	1.18 (0.77–1.83)	0.452
TT	33	18 (54.5)	2.25 (1.30–3.89)	**0.004**	2.02 (1.16–3.54)	**0.014**
rs16949649						
TT	86	27 (31.4)	reference		reference	
TC	152	54 (35.5)	1.20 (0.76–1.90)	0.443	1.12 (0.70–1.80)	0.632
CC	55	21 (38.2)	1.33 (0.75–2.36)	0.323	1.15 (0.64–2.07)	0.639

*P* values in bold were statistically significant.

Abbreviations: HR: hazard ratio; CI: confidence interval.

^a^ Adjusted for age, sex, differentiation, clinical stage, lymphatic metastasis and tumor location.

## Discussion

In this study, we assessed the role of rs2302254 and rs16949649 polymorphisms in the risk of developing CRC and the prognoses of CRC patients. The results indicated that the two genetic variants of *NME1* were not associated with susceptibility to CRC but may be associated with the clinical outcomes of CRC patients. Patients with the rs2302254 TT genotype had a significantly worse prognosis than those with the CC genotype.

Expression of NME1 was shown to be aberrant in a variety of solid tumors (Zhang et al., 2023b; Guo et al., 2022; Bao et al., 2022). However, the underlying mechanism of the regulation of *NME1* expression remains poorly understood. Ouatas et al. (2002) reported that three regions within the *NME1* promoter are involved in differential NME1 expression in breast cancer cells: i.e., a 544 bp AvrII fragment, which consists of an inhibitory element; a 248 bp AvrII–Nhel fragment, which results in increased NME1 expression; and a 195 bp NheI-XbaI fragment, which is responsible for the basal expression level of NME1. Deletion of the 544 bp AvrII fragment, which includes the rs16949649, rs3760468, and rs3760469 polymorphisms, contributes to a 20% increase in *NME1* promoter activity in MCF7 cells, and deletion of the 195 bp NheI-XbaI fragment, which includes the rs2302254 polymorphism, abolishes the promoter activity of *NME1*. In addition, a study of breast cancer cells revealed that minor alleles of the rs2302254 and rs3760468 polymorphisms decreased *NME1* activity (Qu et al., 2008). These data indicated that single nucleotide polymorphisms (rs2302254 and rs16949649) in the promoter region of *NME1* may regulate the expression of NME1 protein.

The association between rs2302254 and risk of cancer has been investigated in many studies. A meta-analysis revealed that this genetic variant was linked to the risk of gastric cancer (Shi et al., 2018). However, no significant relationship was found between the rs2302254 polymorphism and susceptibility to epithelial ovarian cancer, colorectal cancer or breast cancer (Rajagopal et al., 2020; Li et al., 2012; Márquez-González et al., 2024). Similarly, our results also revealed that there was no significant difference in the genotype or allele frequencies of rs2302254 between CRC patients and controls. However, the rs2302254 TT mutant genotype was associated with an increased risk for advanced clinical stage and lymph node metastasis. Furthermore, CRC patients with the homozygous mutant TT genotype of rs2302254 had significantly shorter DFS and OS times than those with the homozygous wild-type CC genotype. This finding was similar to the results of Li et al. (2012), who reported that the rs2302254 TT genotype was related to poor clinical outcomes in ovarian cancer patients. It has been reported that decreased NME1 was associated with higher clinical stage and worse prognosis in patients with CRC (Fu and Chu, 2015). Thus, we speculated that the rs2302254 polymorphism may impact the progression and survival of patients with CRC by reducing the expression of NME1.

Rs16949649 is one of the most heavily studied genetic variants located in the *NME1* promoter region. A previous study revealed that this polymorphism was associated with the incidence and recurrence of invasive pituitary adenoma (Wang and Sang, 2020). Qu et al. (2008) reported that breast cancer patients carrying the rs16949649 C allele had higher cancer-specific mortality than those carrying the wild-type allele. To date, there have been no reports regarding the role of the rs16949649 polymorphism in CRC. Our study suggested that rs16949649 was not associated with susceptibility to CRC or the prognosis of CRC patients. The reason for the discrepancy in these findings may be different effects of rs16949649 on different cancer types. Therefore, further evidence from well-designed studies is needed to clarify the contribution of this *NME1* genetic variant to the risk and survival of patients with carcinomas.

Two main limitations exist in this study. First, the sample size, especially the number of CRC patients followed for more than 5 years, was relatively small. Moreover, the frequency of rs2302254 T/T genotype is very low in the CRC patients. Thus, our findings on the associations of rs2302254 with the clinicopathological characteristics and prognosis of CRC patients should be interpreted with caution. Second, all study participants were recruited from the Fourth Hospital of Hebei Medical University. Therefore, there may be systematic error and selection bias in this study.

In conclusion, our study suggests that the TT mutation genotype of rs2302254 was related to advanced clinical stage, positive lymph node metastasis, and poor clinical outcomes in CRC patients. The rs2302254 polymorphism in the *NME1* promoter might function as a potential molecular marker for predicting the progression and prognosis of CRC. However, larger-scale and multiethnic studies are needed to confirm our conclusions.

## Data Availability

The original contributions presented in the study are included in the article/supplementary material, further inquiries can be directed to the corresponding author.
